# Polar Localization of Virulence-Related Esx-1 Secretion in Mycobacteria

**DOI:** 10.1371/journal.ppat.1000285

**Published:** 2009-01-30

**Authors:** Fredric Carlsson, Shilpa A. Joshi, Linda Rangell, Eric J. Brown

**Affiliations:** 1 Department of Microbial Pathogenesis, Genentech Inc., South San Francisco, California, United States of America; 2 Department of Pathology, Genentech Inc., South San Francisco, California, United States of America; University of Washington, United States of America

## Abstract

The Esx-1 (type VII) secretion system is critical for virulence of both *Mycobacterium tuberculosis* and *Mycobacterium marinum*, and is highly conserved between the two species. Despite its importance, there has been no direct visualization of Esx-1 secretion until now. In *M. marinum*, we show that secretion of Mh3864, a novel Esx-1 substrate that remains partially cell wall–associated after translocation, occurred in polar regions, indicating that Esx-1 secretion takes place in these regions. Analysis of Esx-1 secretion in infected host cells suggested that Esx-1 activity is similarly localized *in vivo*. A core component of the Esx-1 apparatus, Mh3870, also localized to bacterial poles, showing a preference for new poles with active cell wall peptidoglycan (PGN) synthesis. This work demonstrates that the Esx-1 secretion machine localizes to, and is active at, the bacterial poles. Thus, virulence-related protein secretion is localized in mycobacteria, suggesting new potential therapeutic targets, which are urgently needed.

## Author Summary

Mycobacteria represent a major human health problem globally, and there is a pressing need to identify novel processes and mechanisms including therapeutic targets. The Esx-1 secretion system is required for both *Mycobacterium tuberculosis* and *Mycobacterium marinum* to cause disease, and is absent from vaccine strains such as *Mycobacterium bovis* BCG. Esx-1 is functionally conserved between *M. tuberculosis* and the experimentally amenable *M. marinum*, which is increasingly used to study this secretory system. Bacterial cells are spatially highly organized; in particular, pathogenic bacteria may localize virulence-related protein secretion to specific regions within the cell envelope, a feature that is generally believed to be important for virulence. However, it has not been known whether Esx-1 is compartmentalized. Our work represents the first visualization of protein secretion in mycobacteria in general. Specifically, we show that the Esx-1 apparatus localizes to, and is active at, the bacterial poles in *M. marinum*. These findings suggest previously unappreciated mechanisms governing localization of protein secretion in mycobacteria, potentially including new therapeutic targets.

## Introduction

Mycobacteria, and in particular *M. tuberculosis*, represent a major human health problem globally [1]. The Esx-1 secretion system [early secreted antigen 6 kilodaltons (Esat-6) secretion system 1], which is primarily encoded by genes within, and adjacent to, the region of difference 1 (RD1), is a major virulence determinant of both *M. tuberculosis* and *M. marinum*, apparently regulating bacterial spread to host cells [2]–[7]. In *M. tuberulosis* the RD1 locus (*rv3871-rv3879c*) encodes the canonical Esx-1 substrates Cfp-10 and Esat-6, as well as Rv3871 and Rv3877, two of the three core proteins in the secretory apparatus [3],[8]. The third core constituent, Rv3870, is encoded just upstream of RD1 [3],[8], but the Rv3870 protein is not functional in the absence of this locus. Importantly, the RD1 locus is highly conserved between *M. tuberculosis* and *M. marinum* [4],[5],[9], and all Esx-1 deficient mutants analyzed in *M. marinum* thus far have been functionally complemented by their *M. tuberculosis* homologues, demonstrating that the genetic conservation extends to function [5],[10],[11]. Thus, *M. marinum* constitutes a highly relevant system in which to study functional aspects of the Esx-1 secretion system, likely to extend to *M. tuberculosis*.

It is becoming increasingly clear that pathogenic bacteria are able to specifically localize virulence-related secretory systems and protein secretion to distinct compartments within their cell envelopes, and it is generally believed that such localization may be important for virulence [12]–[18]. However, protein secretion has never been visualized in mycobacteria, and it is therefore not known whether secretion in these bacteria is compartmentalized; in particular, there has been no visualization of Esx-1, likely because of technical difficulties arising from the complex and hydrophobic nature of the mycobacterial cell wall. Moreover, analysis of this problem has not been possible because none of the described Esx-1 substrates are known to remain associated with the bacterial surface upon translocation, essentially precluding their use as tools to visualize sites of active Esx-1 secretion. We therefore sought to identify a novel Esx-1 substrate with properties allowing such analysis, and report here that Mh3864 (Marinum homologue of Rv3864; MMAR_5439) is such a protein. Analysis of Mh3864 demonstrated that active Esx-1 secretion occurs in polar regions. Furthermore, using Mh3870 (Marinum homologue of Rv3870; MMAR_5445) as a marker for Esx-1, we show that the secretory apparatus also localizes to the poles. Interestingly, however, the steady-state distribution of Mh3864 in the *M. marinum* cell wall is not strictly polarized, and we propose a mechanism that may account for this feature.

## Results

### Mh3864 is a novel Esx-1 substrate with significant cell wall association

In a transposon mutagenesis screen we identified an Mh3864-insertion mutant by virtue of its smooth colony morphology, which is a common feature of mutants affected in the Esx-1 secretion system (Figure S1A). The Mh3864::tn mutant was deficient in CFP-10 secretion, and exhibited modestly reduced growth in macrophages compared to wild type *M. marinum* (Figure S1B and S1C), suggesting roles for Mh3864 in Esx-1 secretion and virulence.

**Figure 1:**
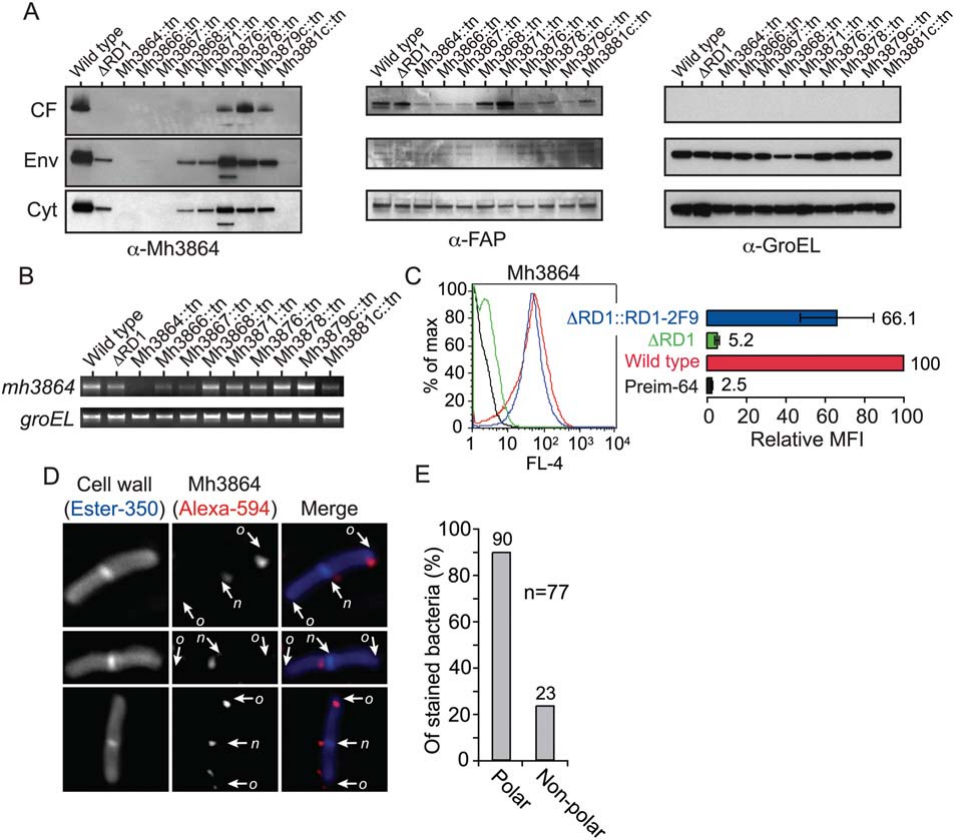
Mh3864 is secreted via Esx-1 in polar regions, and remains partially cell surface–associated after translocation. (A) Indicated *M. marinum* strains were fractionated into culture filtrate (CF), cell envelope (Env; containing cell membrane and cell wall), and cytosolic fractions (Cyt), and analyzed for Mh3864, FAP, and GroEL by immunoblot. (B) RNA from indicated strains was analyzed for *mh3864* and *groEL* transcripts by RT-PCR. No PCR products were obtained when RT-polymerase had been omitted (not shown). (C) Indicated strains were analyzed for reactivity with anti-Mh3864 serum by FACS. As control we analyzed binding of preimmune serum from the same rabbit (preim-64) to wild type. Left panel: Shown is a representative histogram. Right panel: Mean fluorescence intensity (MFI) was normalized to wild type reactivity with anti-Mh3864 Abs. Values are mean+/−SD of three experiments. (D) Localization of newly secreted Mh3864 was analyzed by IF-microscopy on bacteria that had been grown for ≤2 generations after treatment with trypsin (0.2 mg/ml). Mh3864 was probed with anti-Mh3864 serum, and the cell wall was labeled with Ester-350. Shown are representative wild type cells. New poles (i.e. septa) are indicated with *n*, and old poles with *o*. (E) Quantification of the proportion of stained bacteria, from (D), that contained Mh3864 in polar and non-polar regions.

To analyze the subcellular localization of Mh3864 we fractionated *M. marinum* cultures into secreted fraction (culture filtrate; CF), cell envelope fraction (Env) and cytosolic fraction (Cyt) for Western blot analysis using a rabbit antiserum raised against an 89-residue peptide derived from the C-terminal region of Mh3864. This antiserum specifically recognized a ∼40 kDa protein species, corresponding to the expected size of Mh3864, in all three fractions from wild type bacteria (Figure 1A, left panel). No reactivity was observed in Mh3864::tn fractions, demonstrating specificity of the antiserum. In bacteria lacking the entire RD1-region (ΔRD1), Mh3864 was produced but not secreted into the CF, indicating that Mh3864 is a secreted protein, dependent on Esx-1 for its export. Moreover, the specific genetic requirements for Mh3864 secretion were very similar to those previously shown for Cfp-10 and Esat-6, because mutants of Mh3866, Mh3867, Mh3868, Mh3871 and Mh3881c also failed to secrete Mh3864, whereas transposon insertions in genes encoding Mh3876, Mh3878 or Mh3879c did not have this effect (Figure 1A, left panel) [3],[5],[10],[11]. Thus, Mh3864 is an Esx-1 substrate that has significant association with the cell envelope. In mutants that failed to secrete Mh3864, the protein was either completely absent or its cellular concentration reduced, which might be explained by the common finding that stability of Esx-1 constituents and substrates appears to require an intact secretion system [5],[19]. A band of lower molecular weight appeared in the Mh3876::tn strain, which may represent a proteolytic fragment of Mh3864. As controls we analyzed FAP, which is secreted into the CF via the general secretory pathway [20], and GroEL, which is not secreted into the CF (Figure 1A, middle and right panels). These controls indicated that none of the strains were generally deficient in protein secretion, and that there was no nonspecific leakage of cytosolic or envelope material into the CF. Moreover, the Mh3864-encoding gene was transcribed in all strains except Mh3864::tn, suggesting that the influence of Esx-1 on Mh3864 secretion/stability was exerted at the protein level (Figure 1B).

FACS analysis demonstrated that Mh3864 was surface exposed on wild type, but not on ΔRD1 bacteria (Figure 1C). Complementation with the *M. tuberculosis* derived RD1-2F9-cosmid restored surface exposure [2],[21]. Because Mh3864 was produced but not secreted in ΔRD1 bacteria (Figure 1A and 1B), this further indicated that Mh3864 secretion requires Esx-1 and also highlights the functional conservation of this secretory pathway between *M. tuberculosis* and *M. marinum*.

### Active Esx-1 secretion occurs at the bacterial poles

As a fraction of Mh3864 remains surface associated upon secretion, we hypothesized that immunofluorescence (IF) microscopy analysis of newly secreted Mh3864 might allow us to gain insight into the localization of active Esx-1 secretion. To this end we treated bacteria with trypsin, which removed Mh3864 without killing the bacteria, and reinoculated treated cells into broth to allow for protein synthesis. Subsequently, we fluorescently labeled the bacterial cell wall with Ester-350 (Alexa Fluor-350 carboxylic acid, succinimidyl ester) and probed for new Mh3864 using our antiserum (Figure 1D and 1E). No Mh3864 staining was observed on trypsinized bacteria (Figure S2), demonstrating that surface exposed Mh3864 was efficiently removed. Interestingly, after allowing for new protein synthesis in trypsinized cells, Mh3864 appeared primarily at the poles, including both old poles and new poles formed at the division septum (Figure 1D). Analysis of many cells demonstrated that 90% of the stained bacteria had fluorescent foci in polar regions and 23% had foci in non-polar regions (Figure 1E; this adds to >100% because some bacteria had polar and non-polar foci), indicating that Esx-1 secretion occurs primarily at the poles.

### A KasB-deficient mutant strain allows for direct visualization of the Esx-1 apparatus

**Figure 2:**
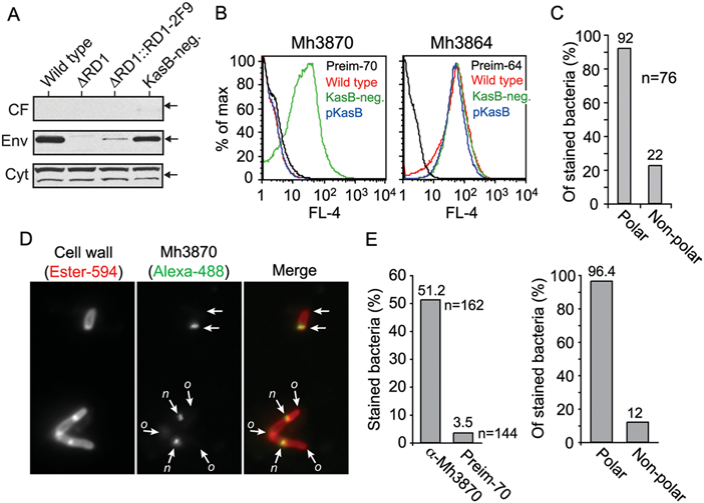
The Esx-1 apparatus can be studied in KasB-negative bacteria, and localizes mainly to polar regions. (A) Anti-Mh3870 serum was used to analyze, by immunoblot, the subcellular localization of Mh3870 in fractions of indicated strains. Arrows indicate ∼75 kDa. (B) Left panel: Anti-Mh3870 serum was used for FACS-analysis of surface exposed Mh3870 on indicated strains. As control we analyzed reactivity between preimmune serum from the same rabbit (preim-70) and KasB-negative bacteria. Right panel: Similar FACS-analysis of Mh3864. Reactivity between wild type bacteria and preim-64 was analyzed as control. Shown are representative FACS-histograms. (C) IF-microscopy analysis of newly secreted Mh3864; quantification of the proportion of KasB-negative bacteria that stained with anti-Mh3864 serum in polar and non-polar regions. (D) Surface distribution of Mh3870 on KasB-negative bacteria was visualized with anti-Mh3870 serum by IF-microscopy. When possible to determine, new poles are indicated with *n*, and old poles with *o*. (E) Left panel: IF-microscopy analysis of many cells indicated that anti-Mh3870 reactivity was specific, and virtually no cells were stained by preim-70. Right panel: Quantification of the proportion of stained KasB-negative bacteria (83 cells) that contained Mh3870 in polar and non-polar regions.

The finding that newly secreted Mh3864 localized to the poles suggested that the Esx-1 apparatus might have a polar distribution in the cell envelope. To analyze the spatial distribution of Esx-1 directly, we generated an antiserum against Mh3870, a membrane-associated component of the Esx-1 secretion apparatus. This antiserum, raised against a 135-residue peptide corresponding to amino acids 334–468, specifically recognized Mh3870 as a ∼75 kDa species in the cell envelope fraction of wild type bacteria (Figure 2A). Mh3870 reactivity was absent in ΔRD1, presumably due to destabilization of the Mh3870 protein in the absence of an intact secretory apparatus [5],[19], and reappeared upon complementation with RD1-2F9. In the cytosolic fraction, the antiserum reacted with two protein species of unknown origin. This nonspecific reactivity was unrelated to RD1 (as it appeared in ΔRD1), and does not affect analysis of the spatial distribution of envelope-associated Mh3870, as these bands were absent from the envelope fractions.

Anti-Mh3870 could not be used to localize Esx-1 on wild type *M. marinum*, because the serum did not react with intact wild type cells (Figure 2B, left panel). Moreover, even affinity purified anti-Mh3870 antibodies were incompatible with methods to label thin-sections for electron microscopy analysis (not shown). However, Mh3870 was accessible to antibodies in a KasB-deficient mutant strain (Figure 2B, left panel), which has a more permeable cell wall [22]. The increased accessibility of Mh3870 in this strain was specifically due to loss of KasB, as trans-complementation with *kasB* (pKasB) eliminated the Mh3870 staining. Previous analysis of the *kasB* mutation has shown that it specifically causes a 2 to 4 carbon reduction in the length of cell wall mycolic acids, which normally are ∼80 carbons long, and a slight change in the mycolate composition. These seemingly small changes cause a drastic increase of cell wall permeability, most likely due to effects in the outer lipid coat of the mycobacterial cell wall where mycolic acids are believed to reside [22].

*In silico* analysis of both Mh3870 and its *M. tuberculosis* homologue Rv3870, which are 90% identical in primary structure, has suggested that these proteins are integral membrane proteins containing AAA-ATPase domains between residues 456 to 665 (see Materials and Methods). The finding that Mh3870 was accessible to antibodies on intact cells (Figure 2B, left panel) represents the first experimental data on the topology of this protein and strongly suggests an extracytoplasmic location for at least some epitopes within residues 334–468. However, our data do not exclude an intracytoplasmic location of the predicted AAA-ATPase domain.

The subcellular localization of Mh3870 was unaffected by absence of KasB (Figure 2A), and the amount of surface exposed Mh3864 was similar in wild type and KasB-negative bacteria (Figure 2B, right panel), indicating that Esx-1 secretion is unaffected by KasB deficiency. Furthermore, IF-microscopy analysis of newly secreted Mh3864 on KasB-negative bacteria (Figure 2C, and Figures S2 and S3) demonstrated similar surface distribution to wild type (Figure 1E), suggesting that *kasB*-inactivation had no effect on the spatial distribution of Esx-1 secretion. Thus, the KasB-mutant could be used to study the localization of Mh3870, as a marker for the Esx-1 apparatus.

### The Esx-1 apparatus localizes to bacterial poles, with a preference for new poles

Strikingly, IF-microscopy demonstrated that Mh3870 localized almost exclusively to the bacterial poles (Figure 2D and 2E). Quantification of a large number of cells indicated that staining was specific (Figure 2E, left panel). ∼96% of stained cells had Mh3870 at a pole, while only ∼12% had non-polar staining (Figure 2E, right panel). Among the polarly stained bacteria, the vast majority (78.3%) were stained in a unipolar fashion mainly at the new bacterial pole (i.e. septum), indicating that Esx-1 localized primarily to this region (Figure S4). Thus, our analysis of Mh3870 and of a newly secreted Esx-1 substrate (Mh3864) strongly suggested that the Esx-1 apparatus localizes to, and is active at, polar regions.

### In infected host cells, the Esx-1 apparatus is active at bacterial poles with actin tail formation

**Figure 3:**
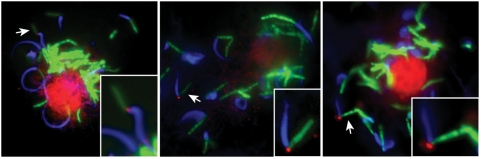
Esx-1 secretion occurs at poles with actin tails in the cytosol of infected host cells. Bone marrow derived mouse macrophages where infected with GFP-expressing wild type *M. marinum* (green) for 24 h, and stained for Mh3864 (red) and actin tails (blue). Mh3864 was visualized using anti-Mh3864 serum followed by Alexa fluor-594 conjugated secondary antibodies, and polymerized actin was visualized with Alexa fluor-350 conjugated phalloidin. All bacteria observed with Mh3864 staining contained Mh3864 at one pole, which was also associated with an actin tail. Shown are three representative infected macrophages. Inlets (lower right corners) represent enlargements of bacteria with Mh3864-staining (indicated with arrows). Of note, unspecific host-nuclear staining was observed with both anti-Mh3864 and preimmune serum (not shown).

To analyze sites of Esx-1 secretion in a milieu more representative of the mycobacterial *in vivo* situation, we visualized Mh3864 localization on wild type *M. marinum* in infected macrophages (Figure 3). While staining of intracellularly growing bacteria was rare, stained cells exhibited a unipolar localization of Mh3864. No staining was observed with preimmune serum, demonstrating specificity (not shown). Thus, in infected host cells, Esx-1 activity is concentrated to one of the bacterial poles, suggesting that polarized Esx-1 secretion is relevant *in vivo*. Because *M. marinum* has previously been shown to form actin tails at one of their poles after reaching the cytosol of infected host cells [23], we were also interested in examining if Esx-1 activity localized to such poles (Figure 3). To this end we used fluorescently conjugated phalloidin, which binds polymerized actin and allows for visualization of actin tails. Indeed, Mh3864 localized to poles with actin tails, indicating that the Esx-1 machine is active at poles that are also competent to induce actin polymerization. Moreover, because the Esx-1 machine localized primarily to new poles (Figure 2D and Figure S4; see also Figure 5A), this also implied that actin polymerization occurs preferentially at new bacterial poles. While Mh3864 itself is not required for actin tail formation (not shown), further studies are warranted to elucidate a possible role for Esx-1, which is required for *M. marinum* to reach the cytosol, in actin tail formation.

### The steady-state surface distribution of Mh3864 is not strictly polarized

**Figure 4:**
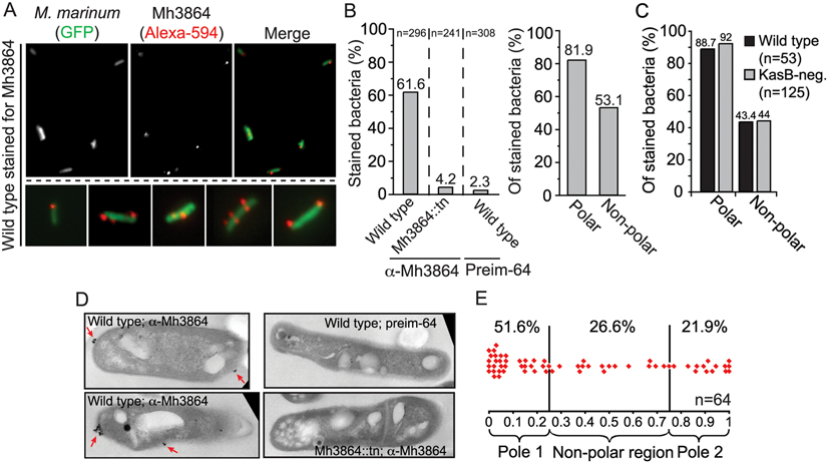
The Mh3864 protein is less polarized in the steady-state. (A) Surface Mh3864 was detected with anti-Mh3864 serum and visualized by IF-microscopy. Shown are representative wild type cells. (B) Left panel: IF-microscopy analysis of many cells indicated that anti-Mh3864 reactivity with wild type was specific; virtually no Mh3864::tn cells labeled with anti-Mh3864 Abs, and almost no wild type bacteria stained with preim-64. Right panel: Quantification of the proportion of stained wild type bacteria that contained Mh3864 in polar and non-polar regions. Data are based on two separate experiments. (C) Similar IF-microscopy analysis of Mh3864 on Ester-594 labeled wild type and KasB-negative bacteria (Mh3864 was detected with Alexa-488 conjugated secondary Abs). (D) Immuno-TEM analysis of Mh3864 surface distribution. Bacterial strains were analyzed with anti-Mh3864 serum or preim-64 as indicated, and IgG-binding was detected with gold-conjugated goat anti-rabbit IgG. No gold particles were observed on controls (right panels). Mh3864 was present in aggregates on the bacterial surface (indicated with red arrows). (E) For each cell, the longest distance between the poles (*D*; bacterial length) and the distance between pole 1 (defined in Methods) and individual gold aggregates (*d*) were measured. The localization of each aggregate is represented as a ratio (*d*/*D*).

IF-microscopy analysis of the steady-state distribution of Mh3864 on GFP-expressing bacteria showed a less polarized distribution than that of newly secreted Mh3864 (Figure 4A and 4B). As expected, wild type bacteria showed specific immunofluorescence, and Mh3864::tn did not (Figure 4B, left panel). Of the stained bacteria, 81.9% had fluorescent foci in polar regions and 53.1% had foci in non-polar regions (Figure 4B, right panel). Similar analysis on non-GFP-expressing wild type and KasB-negative bacteria whose walls had been fluorescently labeled with Ester-594 confirmed this finding and also indicated that the steady-state distribution of Mh3864 was unaffected by KasB-deficiency (Figure 4C and Figure S5). Compared to data in figure 4B, there was a ∼10% distribution-shift towards polar regions in the current analysis, which likely can be explained by improved visualization of septal regions/new poles by Ester-594. Taken together, steady-state analysis of Mh3864 (Figure 4C) indicated a largely polarized surface distribution, but with a ∼2-fold increase of staining in non-polar regions as compared to newly secreted Mh3864 (Figure 1E and Figure 2C). Moreover, the steady-state distributions of Mh3870 and Mh3864 were partially distinct; Mh3870 localized to non-polar regions in merely 12% of stained cells (Figure 2E, right panel), whereas Mh3864 did so in ∼44% of cells (Figure 4C). These findings implied that, during bacterial growth, at least some Mh3864 protein moved to non-polar regions after translocation to the cell wall at the poles.

As Mh3864 was present in distinct aggregates on the bacterial surface, and because immunofluorescence may exaggerate the signal from molecular aggregates, we analyzed the steady-state distribution of surface Mh3864 by immuno transmission electron microscopy (Figure 4D). This analysis confirmed the focal appearance of Mh3864 on the bacterial surface, and allowed us to analyze the distribution in more detail; ∼73% of all gold aggregates localized to polar regions while ∼27% were non-polarly distributed (Figure 4E).

### The Esx-1 apparatus localizes mainly to new poles with active cell wall growth

**Figure 5:**
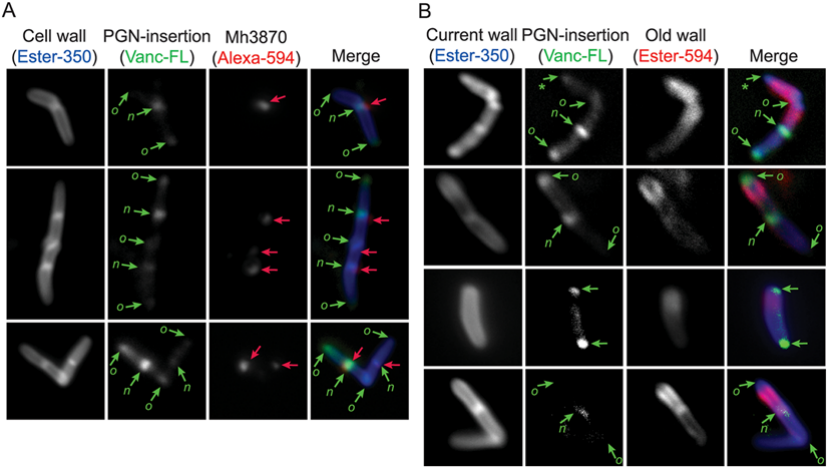
Esx-1 secretion occurs in polar regions, from where the cell wall PGN layer grows. (A) IF-microscopy analysis of co-localization between Mh3870 and Vanc-FL, which labels sites of PGN-insertion into the cell wall. For visualization of PGN-insertion sites, KasB-negative bacteria were incubated with a 1:1 mixture of vancomycin:Vanc-FL (1 µg/ml final concentration) for 4 h at 30°C. Subsequently the bacterial surface was labeled with Ester-350, and Mh3870 (red arrows) was probed with anti-Mh3870 serum. New and old poles are indicated with *n* and *o*, respectively. (B) KasB-negative bacteria were prepared for IF-microscopical analysis, as explained in the text, to analyze migration of old wall. When determinable, new and old poles are indicated as described above, and those likely to represent even one generation older poles are indicated with an asterisk.

In order to study Esx-1 localization in more detail, and to gain insights into the cell wall properties at these sites, we analyzed co-localization between Mh3870 and fluorescently labeled vancomycin (Vanc-FL) on Ester-350 labeled bacteria (Figure 5A). Vancomycin binds to the pentapeptide precursor (Lipid II) during the production of cell wall PGN, and it is well established that Vanc-FL can be used to probe sites of PGN-insertion into the preexisting cell wall [24]–[27]. For wild type *M. marinum* the MIC value of vancomycin was exceedingly high (≥80 µg/ml), and we were unable to obtain Vanc-FL staining in these bacteria (not shown). However, KasB-negative bacteria were inhibited at a much lower concentration of vancomycin (≤1 µg/ml), and also stained efficiently with Vanc-FL (Figure 5A). This analysis demonstrated that *M. marinum* inserts new cell wall PGN at both new poles/septa and old poles [27], indicating that both poles may represent dynamic and active regions. However, new poles often stained more intensely with Vanc-FL than old poles, suggesting that new poles might represent more active sites of cell wall growth. Colocalization of Mh3870 with Vanc-FL at septa (Figure 5A) confirmed that Mh3870 localized mainly to new poles, indicating that Esx-1 localizes primarily to a region of active cell wall turnover.

Because our IF-microscopy analysis suggested that some Mh3864 protein might move from its polar site of secretion during bacterial growth, we hypothesized that Mh3864 might move with the cell wall towards non-polar regions, as it was being pushed from the poles by continuous insertion of new PGN. To test if the wall migrates during bacterial growth, we stained bacteria with Ester-594, allowed them to grow for 3 generations, incubated the cultures with Vanc-FL, and finally stained the bacteria with Ester-350 (Figure 5B). This allowed us to visualize “old wall” (Ester-594), sites of PGN-insertion (Vanc-FL), and the “current wall” (Ester-350). This analysis demonstrated that old wall was absent from polar regions of PGN-insertion, whereas staining of the current wall covered the entire bacterial surface (Figure 5B), indicating that the old wall had indeed migrated towards non-polar regions during bacterial growth.

## Discussion

Our data demonstrate that the Esx-1 secretion apparatus localizes to bacterial poles, primarily to new poles with active cell wall synthesis. These findings were made possible by the use of a mutant strain with a more permeable outer lipid coat (KasB-neg.), which allowed penetration by antibodies and fluorescent probes. Importantly, analysis of a novel Esx-1 substrate that remains partially cell wall-associated (Mh3864) showed that active Esx-1 secretion occurs primarily at bacterial poles. Interestingly, Mh3864 also localized to bacterial poles in infected macrophages, suggesting that polarized Esx-1 secretion is relevant in the context of an infected host. Thus, the Esx-1 apparatus localizes to, and is active at, the bacterial poles.

The role of Mh3864 homologues in mycobacterial virulence remains unclear. Analysis in *M. tuberculosis* indicates that Rv3864-deficient bacteria are attenuated *in vivo* [28], whereas its homologue in *Mycobacterium leprae* (ML0058c) is a pseudogene and studies in *Mycobacterium microti* speak against a required role for Rv3864 in virulence [29]. Moreover, a study in *M. tuberculosis* has shown that Rv3616c (EspA), a homologue of Rv3864, is an Esx-1 substrate required for virulence [19]. Thus, although there has been no systematic comparison of the functions of Rv3616c and Rv3864 or their orthologues in any mycobacterial species, the apparently conflicting data regarding the role of Rv3864 in virulence might possibly be explained by redundancy, in at least some mycobacterial species. Our work identifies Mh3864 as the first bona fide Esx-1 substrate that remains partially cell surface-associated, and accessible to antibodies on intact wild type cells, and also suggests a role for Mh3864 in *M. marinum* virulence. It is therefore intriguing to speculate that its functional homologue in *M. tuberculosis* might represent a potential vaccine candidate.

Specialized secretion systems, such as Esx-1, are common among pathogenic bacteria; for example, type III secretion is critical for virulence of Salmonella, Shigella and Yersinia [30], and type IV secretion is similarly required for Helicobacter, Legionella and Agrobacterium [31]. Interestingly, the type III and type IV secretion machines may be specifically active at bacterial poles [15],[18], implying that polar localization of virulence related protein secretion is a common feature in pathogens. However, it remains unknown if polar localization of these well-studied secretory systems is required for virulence, possibly because the molecular mechanisms of localization are intimately connected to proficient secretion. Concerning mycobacteria, identification of the genetic requirements and the mechanisms governing Esx-1 localization will open the path to address this important question.

These studies also allowed us to propose a link between cell wall growth and Esx-1 localization. According to this model Mh3864 is secreted via Esx-1 in polar regions with active PGN biosynthesis. As new PGN is inserted at the poles it may push the existing cell wall PGN layer, including associated Mh3864, towards non-polar regions, explaining why some Mh3864 localizes to non-polar regions during steady-state growth. However, Mh3870 remains polarized at steady-state, emphasizing the distinct behavior of the Esx-1 secretion machine. Interestingly, this model, which takes into account both the site of secretion and the dynamics of cell wall growth, is in principle similar to findings in *Streptococcus pyogenes* and *Listeria monocytogenes* [16],[25],[32],[33]. Thus, with regard to the steady-state distribution of wall-associated surface proteins it appears that a functional relationship between site of secretion and the dynamics of cell wall growth might be of general importance in Gram-positive bacteria, including mycobacteria.

Identification of the mechanisms governing Esx-1 localization will be of great interest since they may be required for mycobacterial virulence, and amenable to therapeutic intervention.

## Materials and Methods

### Strains and culture conditions

Wild type *M. marinum* M-strain and an isogenic deletion mutant lacking RD1 (ΔRD1) has been described previously [34], as well as insertional transposon mutants of Mh3866, Mh3867, Mh3868, Mh3871, Mh3876, Mh3878, Mh3879c and Mh3881c [5],[10]. An insertional transposon mutant of KasB, and its trans-complement (pKasB) has been described [22]. ΔRD1 was complemented with RD1-2F9 by integration of this cosmid into the chromosomal attB-site [2],[21]. A wild type *M. marinum* M-strain expressing *gfp* that has been integrated into the attB-site has been previously described [34]. *M. marinum* strains were grown in Middlebrook 7H9-broth (Difco) supplemented with 0.2% glycerol, 0.05% Tween 80, and 10% albumin-dextrose-catalase enrichment, or on 7H10 agar (Difco) supplemented with 0.5% glycerol and 10% oleic acid-albumin-dextrose-catalase enrichment. Cultures were supplemented with antibiotics as appropriate. For fractionation of *M. marinum* cultures, they were grown in Sauton's defined medium (Teknova).

### Identification of an Mh3864 transposon mutant

Wild type *M. marinum* expressing *gfp* from the chromosome was subjected to an M^4^ (*mariner* transposon mutagenesis in *M. marinum*) mutagenesis screen as previously described in detail [35]. The site of transposon insertion was determined as described [35]. Finally, PCR analysis and DNA sequencing demonstrated that the transposon was inserted in an inverted position immediately down-stream of nucleotide 252 in the Mh3864-encoding gene, corresponding to a truncation after amino acid 84 in Mh3864.

### Fractionation of *M. marinum* cultures

Strains were grown to mid-log phase (OD_600_ = 0.7+/−0.2) in 7H9. The bacteria were washed extensively in Sauton's minimal medium, and inoculated into 20 ml (final volume) of this medium. Short-term cultures were collected ∼48 h after inoculation. Bacteria were pelleted by centrifugation, and the supernatant was filtered through a 0.2 µm filter (culture filtrate). After a ∼65-fold concentration of the culture filtrates using Vivaspin 15R (2.000 MWCO; Sartorius Biolab), the final volume was determined for later normalization. The bacterial pellet was weighed for normalization purposes, and subsequently resuspended in 2 ml fractionation buffer (100 mM HEPES, pH 7.5; 300 mM KCl; 10% glycerol; 10 mM MgCl_2_; 1 mM DTT; 0.01% Tween 80) supplemented with complete, EDTA-free, protease inhibitor coctail (Roche). Bacterial cell lysates were prepared by bead beating at 4°C, and centrifugated at 3000×g for 10 min to pellet glass beads and remaining intact bacteria. The cell envelope fraction, containing both the cell membrane and the cell wall, was collected by subjecting the supernatant to ultracentrifugation (100.000×g) for 1 h, and resuspended in 0.4 ml fractionation buffer. The supernatant from the ultracentrifugation was collected as cytosolic fraction, and its volume determined. For Western blot analysis of fractions, loading was normalized to the weights of the original bacterial pellets, and samples were separated by SDS-PAGE, using 4–20% gradient gels (Bio-Rad). Membranes were developed with West Pico (Pierce).

### Reverse transcription PCR-analysis

RNA was purified from mid-log phase *M. marinum* cultures (OD_600_ = 0.7+/−0.2) using RNeasy Mini Kit (Qiagen), essentially as described by the manufacturers. However, bacterial lysates were first prepared by bead beating as described above, and we included an additional step of DNase I (New England Biolabs) treatment to ensure degradation of chromosomal DNA. C-DNA for *mh3864* and *groEL* were generated in the same tube by RT-PCR on 1 µg RNA using reverse primers *mh3864*_3prR (5′-ttcgtcgtcttccttcttgtcgct-3′) and *groEL*_3prR (5′-tctcggtggtcagcaccatacgtg-3′), respectively. In control tubes, RT-polymerase was omitted; no PCR products (see below) were obtained when these controls were used as template, demonstrating absence of contaminating chromosomal DNA (not shown). Generated c-DNA was used as template for PCR-analysis of *mh3864* and *groEL* using primer pairs *mh3864*_5prF (5′-gctcttcaaaggaatcgccgacaa-3′) and *mh3864*_3prR, and groEL_5prF (5′-tgagcaagctgattgagtacgacg-3′) and *groEL*_3prR, respectively. Equal amounts were loaded for gel analysis using 1% agarose gels.

### Generation of antiserum to Mh3864 and Mh3870

To generate a rabbit antiserum against Mh3864, an 89-residue peptide corresponding to amino acids 330–418 was cloned into pGEX-KG as a translational GST-fusion. *Escherichia coli* BL21 Codon Plus (Stratagene) harboring the construct was grown at 37°C and expression of the fusion peptide was induced with 1 mM IPTG. After purification on a glutathione sepharose column (GE Healthcare) followed by an S300 column (GE Healthcare), the GST-tag was separated off by overnight thrombin digestion that cleaved a thrombin cleavage site located between the tag and the Mh3864 peptide. The Mh3864 peptide was subsequently purified through a glutathione sepharose column followed by an S200 column (GE Healthcare). A 135-residue peptide corresponding to amino acids 334–468 of Mh3870 was expressed in a similar manner. However, the Mh3870 fusion peptide was insoluble and extracted from inclusion bodies by 6 M Guanidine Hydrochloride, and refolded overnight in TRIS/HCl buffer containing 3.5 M urea. After refolding the urea concentration was reduced to 1 M by buffer exchange. The GST-tag was removed by thrombin digestion as described above, and the Mh3870 peptide was purified on a RP C4 column (W.R. GRACE). Finally, the purified Mh3864 and Mh3870 peptides were confirmed by mass spectrometry analysis. Rabbits were immunized with 200 µg of the purified peptides using TiterMax (TiterMax USA, Inc.) as adjuvance, and subsequently similarly boosted with 100 µg antigen. The use of TiterMax was critical since it provided a good immune response, but does not contain any mycobacterial components. For both antigens the rabbits were also bleed prior to immunization in order to obtain relevant preimmune controls, and Western blot analysis of *M. marinum* fractions indicated lack of reactivity for both of these preimmune sera (not shown).

### Probes and antibodies

Carboxylic acid, succinimidyl esters conjugated to either Alexa fluor-350 or 594 (Ester-350 or Ester-594), and Bodipy-conjugated vancomycin (Vanc-FL) were from Molecular probes. For IF-microscopy and FACS-analysis, our generated rabbit antisera against Mh3864 and Mh3870 were used at 1:500 and 1:200, respectively. For IF-microscopy, binding of rabbit IgG was detected with goat anti-rabbit IgG (H+L) antibodies conjugated with either Alexa fluor-488 or 594 (Molecular Probes), diluted to 1:250. For FACS-analysis rabbit IgG-binding was detected with an allophycocyanin (APC)-conjugated affinity purified F(ab')_2_ fragment donkey anti-rabbit IgG (H+L) (Jackson ImmunoResearch Laboratories), at 1:100. Gold (6 nm)-conjugated F(ab')_2_ goat anti-rabbit IgG (Electron Microscopy Sciences) was used at 1:200 for electron microscopical detection of bound rabbit IgG. For Western blotting, monoclonal mouse anti-GroEL Abs (Colorado state) were used at 1:50, and polyclonal rabbit anti-FAP serum (Colorado state) was used at 1:15000. Rabbit antisera against Mh3864 and Mh3870 were used at 1:5000 and 1:2000, respectively. Peoxidase-conjugated affinity purified F(ab')_2_ fragment donkey anti-rabbit IgG (H+L) (Jackson ImmunoResearch Laboratories) and peroxidase-conjugated affinity purified F(ab')_2_ fragment donkey anti-mouse IgG (H+L) (Jackson ImmunoResearch Laboratories) were used at 1:5000 for detection of rabbit and mouse IgG, respectively.

### Preparation of bacteria for microscopic and FACS analysis

Bacteria were grown to mid-log phase (OD_600_ = 0.7+/−0.2) in 7H9-broth, and collected by centrifugation. Cells were washed in Tris-buffered saline supplemented with 0.05% Tween-20 (TBST), and needled twice through a 26G1/2 needle (Becton Dickinson) to disrupt bacterial aggregates. Aggregates were pelleted by two separate centrifugation steps (2000 rpm, 1 min), where the supernatants, enriched for single cell bacteria, were transferred to new tubes. The bacterial concentration was determined using a hemacytometer, and suspensions were diluted to a final concentration of ∼10^8^ bacteria/ml.

If the bacteria were to be stained with fluorescently conjugated Carboxylic acid, succinimidyl ester, they were prepared in phosphate buffered saline (PBS), and labeled as instructed by the manufacturer. After labeling, the bacteria were washed twice with TBST to inactivate unbound ester compounds.

### Immuno fluorescence microscopic analysis

1 ml of bacterial suspensions, prepared as described above, were pelleted and resuspended in 0.1 ml TBST containing indicated serum/antibodies. After incubation at room temperature (RT) for 1 h with agitation, the suspensions were washed twice with TBST. For IF-microscopy the bacteria were then similarly incubated with the appropriate fluorescently conjugated secondary antibody. Upon washing the bacteria were mounted with ProLong antifade (Molecular Probes) onto glass cover slips and analyzed with Axioplan 2 Zeis microscope using a 100× objective. For scoring staining as polar, the fluorescence had to be at, or immediately adjacent to, a pole.

For visualization of Mh3864 and actin tails on *M. marinum* in infected macrophages, infected cells were washed once in PBS and fixed with 4% PFA for 20 min. Fixed cells were permeabilized with 0.1% Triton X (Pierce) for 4 min, and subsequently washed 3 times with PBS. Mh3864 was detected by incubation with anti-Mh3864 serum (1/100 in PBS supplemented with 1% BSA) for 1 h. After washing, a fluorescently conjugated secondary antibody was added for visualization of IgG-binding, and Alexa Fluor-350 conjugated phalloidin (Invitrogen) was added for visualization of polymerized actin. After 1 h incubation, cells were washed 3 times in PBS, and mounted for IF-microscopy analysis.

### FACS analysis

Bacteria were prepared as described for IF-microscopy, except that IgG-binding was detected with APC-conjugated secondary antibodies. Samples were run on a FACSCalibur (BD Biosciences), and data was analyzed using FlowJo (Tree Star Inc.).

### Immuno transmission electron microscopy analysis

Bacterial cells were prepared as described for IF-microscopy, but we used a gold (6 nm) conjugated secondary antibody for detection of bound IgG. Cells were fixed in 1/2 Karnovski's (2% paraformaldehyde and 2.5% glutaraldehyde in 0.1 M sodium cacodylate buffer) for 1 h, and then washed in 0.1 M sodium cacodylate buffer and post-fixed in 1% aqueous osmium textroxide for 1 h. Samples were subsequently dehydrated through a series of ethanol, followed by propylene oxide and embedded in Eponate 12 (Ted Pella). Thin sections were cut on a Reichert Ultracut E, stained with 1% uranyl acetate and 0.1% lead citrate, and examined in a Philips CM12 electron microscope. Images were captured with a GATAN Retractable Multiscan digital camera. For quantitative analysis of localization of gold aggregates, the localization of each aggregate is represented as a ratio (*d*/*D*); the distance between pole 1 (defined below) and individual aggregates of gold particles (*d*) was divided by the longest distance between the two bacterial poles (*D*). If gold particles were observed in only one polar region, that pole is pole 1. If gold particles were observed in both polar regions, the pole with more gold particles is pole 1. If gold particles were observed in only non-polar regions, the pole to the left in the picture is pole 1. Each polar region includes 25% of the total bacterial length.

### Trypsination of bacteria

For analysis of newly secreted Mh3864, bacteria were trypsinized essentially as described previously [17],[32]. In brief, mid-log cultures were washed twice with PBS, resuspended in PBS (untreated control) or PBS supplemented with 0.2 mg/ml trypsin (Sigma), and incubated at 37°C for 1 h with agitation. As controls, untreated and trypsinized cells were washed twice with TBST supplemented with 0.02% azide (TBSTA) and immediately probed for Mh3864 as described above. For analysis of newly secreted Mh3864, cells were washed twice with PBS, resuspended in 10 ml pre-warmed 7H9-medium, and grown at 32°C for ≤2 generations as measured by optical density. Finally these cells were fluorescently labeled with Ester-350 and similarly probed for Mh3864.

### Macrophage infections

Bone marrow derived macrophages (BMDM) were obtained and cultured from 129/SVJ mice as previously described [23]. BMDMs were grown on glass cover slips and infected with GFP-expressing *M. marinum* at a MOI of 5, essentially as described [23]. At 24 h post infection, samples were stained for Mh3864 and actin tails as described above. Finally, samples we mounted with ProLong antifade for microscopical analysis.

### In silico analysis

The ExPASy Proteomics MotifScan-tool (http://myhits.isb-sib.ch/cgi-bin/motif_scan) was used to analyze the primary sequences of Mh3870 (MMAR_5445) and Rv3870, which were retrieved from MarinoList (http://genolist.pasteur.fr/ MarinoList/) and TubercuList (http://genolist.pasteur.fr/ TubercuList/), respectively.

## Supporting Information

Figure S1 Identification of an Mh3864-insertional transposon mutant. (A) Left panel: Wild type *M. marinum* exhibits rough colony morphology. Right panel: The Mh3864::tn mutant exhibits smooth colony morphology, which is common to mutants affected in Esx-1 secretion. (B) Left panel: The Mh3864::tn mutant is unable to secrete Cfp-10, which is accumulated in the cytosol, suggesting a role for Mh3864 in Esx-1 secretion. ΔRD1 was analyzed as control. Right panel: Ag85 is secreted via the general secretory pathway (Sec), and is unaffected by Mh3864-inactivation. Shown is representative data from three separate experiments. (C) Bone marrow derived macrophages from 129/SVJ mice were infected (MOI = 3) with *M. marinum*, as indicated. The Mh3864::tn mutant shows reduced growth in macrophages as compared to wild type bacteria. As is commonly seen for mutants of individual genes encoding Esx-1 members and/or substrates, the growth reduction is not as severe as for the ΔRD1 mutant (4). Data representative of three separate experiments.
(8.33 MB EPS)

Figure S2 Trypsin removes surface Mh3864. Wild type and KasB-negative bacteria were treated with 0.2 mg/ml trypsin as described in Materials and Methods. Untreated control and trypsin treated bacteria were probed for Mh3864 using anti-Mh3864 serum, and analyzed by IF-microscopy. Upper panels: Untreated bacteria were analyzed as control and stained with anti-Mh3864 serum. Lower panels: Virtually no trypsin treated bacteria stained with the anti-Mh3864 serum, indicating that trypsin efficiently removed surface Mh3864 protein.
(4.44 MB EPS)

Figure S3 Localization of newly secreted Mh3864 on KasB-negative bacteria. KasB-negative bacteria were trypsinized and reinoculated as described in Materials and Methods. Subsequently the cell wall was labeled with Ester-350, and Mh3864 was probed for using anti-Mh3864 serum (detected with Alexa-594 conjugated secondary Abs). KasB-negative bacteria showed similar surface localization of newly secreted Mh3864 as wild type bacteria. For quantitative data, see Figure 2C. Shown here are representative cells.
(0.57 MB EPS)

Figure S4 Detailed analysis of Mh3870 localization on KasB-negative bacteria. The cell wall of KasB-negative bacteria was labeled with Ester-594, and Mh3870 was localized using anti-Mh3870 serum, detected with Alexa-488 conjugated secondary Ab. (A) Only five principal patterns of localization are possible: unipolar, nonpolar, unipolar and nonpolar, bipolar, and bipolar and nonpolar. Analysis of 83 bacterial cells stained for Mh3870 indicated that 78.3% were stained exclusively in a unipolar fashion. (B) To determine which pole (new or old) Mh3870 localized to among the unipolarly stained cells, only cells allowing distinction between new and old poles could be used (i.e. cells with a clearly visible division septum/new pole as illustrated in top panels. New and old poles are indicated with *n* and *o*, respectively). This caused the exclusion of many unipolarly stained cells, which did not allow such determination because of lack of visible septum (as illustrated in lower panels). Quantification of 25 bacterial cells where new and old poles could be identified indicated that 92% of unipolarly stained bacteria contained Mh3870 at the new pole (bottom graph).
(0.78 MB EPS)

Figure S5 Steady-state distribution of Mh3864 on KasB-negative bacteria. The cell wall of KasB-negative bacteria was labeled with Ester-594, and Mh3864 was probed for using anti-Mh3864 serum (detected with Alexa-488 conjugated secondary Abs), and showed similar surface distribution as on wild type bacteria. For quantitative data, see Figure 4C. Shown here are representative cells.
(0.55 MB EPS)

## References

[1] World Health Organization (2008). WHO Report 2008 Global Tuberculosis Control.. http://www.who.int/tb/publications/global_report/2008/chapter_1/en/index3.html.

[2] Pym AS, Brodin P, Brosch R, Huerre M, Cole ST (2002). Loss of RD1 contributed to the attenuation of the live tuberculosis vaccines Mycobacterium bovis BCG and Mycobacterium microti.. Mol Microbiol.

[3] Stanley SA, Raghavan S, Hwang WW, Cox JS (2003). Acute infection and macrophage subversion by Mycobacterium tuberculosis require a specialized secretion system.. Proc Natl Acad Sci U S A.

[4] Volkman HE, Clay H, Beery D, Chang JC, Sherman DR (2004). Tuberculous granuloma formation is enhanced by a mycobacterium virulence determinant.. PLoS Biol.

[5] Gao LY, Guo S, McLaughlin B, Morisaki H, Engel JN (2004). A mycobacterial virulence gene cluster extending RD1 is required for cytolysis, bacterial spreading and ESAT-6 secretion.. Mol Microbiol.

[6] Cosma CL, Sherman DR, Ramakrishnan L (2003). The secret lives of the pathogenic mycobacteria.. Annu Rev Microbiol.

[7] Guinn KM, Hickey MJ, Mathur SK, Zakel KL, Grotzke JE (2004). Individual RD1-region genes are required for export of ESAT-6/CFP-10 and for virulence of Mycobacterium tuberculosis.. Mol Microbiol.

[8] Abdallah AM, Gey van Pittius NC, Champion PA, Cox J, Luirink J (2007). Type VII secretion—mycobacteria show the way.. Nat Rev Microbiol.

[9] Stinear TP, Seemann T, Harrison PF, Jenkin GA, Davies JK (2008). Insights from the complete genome sequence of Mycobacterium marinum on the evolution of Mycobacterium tuberculosis.. Genome Res.

[10] McLaughlin B, Chon JS, MacGurn JA, Carlsson F, Cheng TL (2007). A mycobacterium ESX-1-secreted virulence factor with unique requirements for export.. PLoS Pathog.

[11] Tan T, Lee WL, Alexander DC, Grinstein S, Liu J (2006). The ESAT-6/CFP-10 secretion system of Mycobacterium marinum modulates phagosome maturation.. Cell Microbiol.

[12] Scott ME, Dossani ZY, Sandkvist M (2001). Directed polar secretion of protease from single cells of Vibrio cholerae via the type II secretion pathway.. Proc Natl Acad Sci U S A.

[13] Rosch J, Caparon M (2004). A microdomain for protein secretion in Gram-positive bacteria.. Science.

[14] Christie PJ, Atmakuri K, Krishnamoorthy V, Jakubowski S, Cascales E (2005). Biogenesis, architecture, and function of bacterial type IV secretion systems.. Annu Rev Microbiol.

[15] Judd PK, Kumar RB, Das A (2005). Spatial location and requirements for the assembly of the Agrobacterium tumefaciens type IV secretion apparatus.. Proc Natl Acad Sci U S A.

[16] Carlsson F, Stalhammar-Carlemalm M, Flardh K, Sandin C, Carlemalm E (2006). Signal sequence directs localized secretion of bacterial surface proteins.. Nature.

[17] DeDent AC, McAdow M, Schneewind O (2007). Distribution of protein A on the surface of Staphylococcus aureus.. J Bacteriol.

[18] Jaumouille V, Francetic O, Sansonetti PJ, Tran Van Nhieu G (2008). Cytoplasmic targeting of IpaC to the bacterial pole directs polar type III secretion in Shigella.. Embo J.

[19] Fortune SM, Jaeger A, Sarracino DA, Chase MR, Sassetti CM (2005). Mutually dependent secretion of proteins required for mycobacterial virulence.. Proc Natl Acad Sci U S A.

[20] Ratliff TL, McCarthy R, Telle WB, Brown EJ (1993). Purification of a mycobacterial adhesin for fibronectin.. Infect Immun.

[21] Koo IC, Wang C, Raghavan S, Morisaki JH, Cox JS (2008). ESX-1-dependent cytolysis in lysosome secretion and inflammasome activation during mycobacterial infection.. Cell Microbiol.

[22] Gao LY, Laval F, Lawson EH, Groger RK, Woodruff A (2003). Requirement for kasB in Mycobacterium mycolic acid biosynthesis, cell wall impermeability and intracellular survival: implications for therapy.. Mol Microbiol.

[23] Stamm LM, Morisaki JH, Gao LY, Jeng RL, McDonald KL (2003). Mycobacterium marinum escapes from phagosomes and is propelled by actin-based motility.. J Exp Med.

[24] Tiyanont K, Doan T, Lazarus MB, Fang X, Rudner DZ (2006). Imaging peptidoglycan biosynthesis in Bacillus subtilis with fluorescent antibiotics.. Proc Natl Acad Sci U S A.

[25] Rafelski SM, Theriot JA (2006). Mechanism of polarization of Listeria monocytogenes surface protein ActA.. Mol Microbiol.

[26] Daniel RA, Errington J (2003). Control of cell morphogenesis in bacteria: two distinct ways to make a rod-shaped cell.. Cell.

[27] Thanky NR, Young DB, Robertson BD (2007). Unusual features of the cell cycle in mycobacteria: polar-restricted growth and the snapping-model of cell division.. Tuberculosis (Edinb).

[28] Sassetti CM, Rubin EJ (2003). Genetic requirements for mycobacterial survival during infection.. Proc Natl Acad Sci U S A.

[29] Brodin P, Majlessi L, Marsollier L, de Jonge MI, Bottai D (2006). Dissection of ESAT-6 system 1 of Mycobacterium tuberculosis and impact on immunogenicity and virulence.. Infect Immun.

[30] Coburn B, Sekirov I, Finlay BB (2007). Type III secretion systems and disease.. Clin Microbiol Rev.

[31] Backert S, Meyer TF (2006). Type IV secretion systems and their effectors in bacterial pathogenesis.. Curr Opin Microbiol.

[32] Swanson J, Hsu KC, Gotschlich EC (1969). Electron microscopic studies on streptococci. I. M antigen.. J Exp Med.

[33] Cole RM, Hahn JJ (1962). Cell wall replication in Streptococcus pyogenes.. Science.

[34] Cosma CL, Humbert O, Ramakrishnan L (2004). Superinfecting mycobacteria home to established tuberculous granulomas.. Nat Immunol.

[35] Gao LY, Groger R, Cox JS, Beverley SM, Lawson EH (2003). Transposon mutagenesis of Mycobacterium marinum identifies a locus linking pigmentation and intracellular survival.. Infect Immun.

